# Effects of Chailong Jieyu Pill on Behavior, Monoamine Neurotransmitters, and Corticosteroid Receptors in a Rat Model of Anxiety Disorder

**DOI:** 10.1155/2018/5489215

**Published:** 2018-05-31

**Authors:** Guang-kui Feng, Xian-jun Ma, Yin-yi Chen, Guang-rong Bian, Chao Yang, Bao-dong Gu

**Affiliations:** ^1^Department of Encephalopathy, Lianyungang Affiliated Hospital, Nanjing University of Chinese Medicine, Lianyungang 222004, China; ^2^Department of Rehabilitation, Wuhan No. 1 Hospital, Wuhan 430000, China

## Abstract

Chailong Jieyu Pill (CJP) is composed of* Radix Bupleuri, Radix Scutellariae, Rhizoma Pinelliae Preparata, Radix Codonopsis, Radix Glycyrrhizae preparata*, keel,* Concha Ostreae, Concha Margaritifera Usta, Rhizoma Zingiberis Recens*, and* Fructus Jujubae*. CJP has shown good clinical effects on improving anxiety disorders. However, as the mechanism underlying such benefits remains unclear, the aim of this study was to investigate the mechanism of action for CJP on anxiety-related behaviors in a rat model of anxiety disorder. After establishing a rat model of anxiety disorder using uncertain empty bottle stimulation, rats were divided into control, model, citalopram, low-dose CJP, and high-dose CJP groups. After 1 month of administration, effects of treatments on rat appearance, body weight, and open-field test scores were observed. In addition, hippocampal monoamine neurotransmitter (5-hydroxytryptamine, dopamine, and norepinephrine) contents were measured with an enzyme-linked immunosorbent assay, and mRNA expression of mineralocorticoid receptor (MR) and glucocorticoid receptor (GR) were measured with reverse transcription-polymerase chain reaction. CJP increased rat weight, and this effect was increased in the high-dose CJP group compared with the citalopram group (*P* < 0.05). CJP also elevated open-field test scores compared with the citalopram group (*P* < 0.05). While CJP decreased monoamine neurotransmitter contents in rat hippocampus, the regulatory effect of CJP on 5-hydroxytryptamine was reduced compared with citalopram (*P* < 0.01). CJP upregulated GR mRNA expression in both low-dose (*P* < 0.05) and high-dose (*P* < 0.01) CJP groups, but only the latter significantly downregulated MR mRNA expression and showed enhanced effects compared with citalopram (*P* < 0.05). Thus, CJP likely exerted its significant antianxiety effect by diminishing monoamine neurotransmitters and regulating mRNA expression of MR and GR in the hippocampus of our rat model of anxiety disorder.

## 1. Introduction

The incidence of anxiety disorder is increasing yearly, with a lifetime prevalence of anxiety disorder of approximately 4.1% in China [1] and up to 30% in Europe [2]. Anxiety disorder is mainly induced by emotional discomfort, depression, and stagnation of* qi*. Indeed, stagnation of liver* qi* and* qi* stagnation are key for pathogenesis. Dispersing stagnated liver* qi* to relieve* qi* stagnation is the basic principle of treatment. Our hospital preparation, Chailong Jieyu Pill (CJP), was approved and licensed by Jiangsu Province Hospital in 2004. CJP can disperse depressed liver energy, regulate vital energy, clear liver heat, invigorate the spleen to remove phlegm, and suppress, tranquilize, and calm adverse-rising energy. Through 10 years of clinical practice, we have discovered that CJP elicits good clinical effects for improving anxiety and insomnia. This includes a study using a placebo control, whereby 61 anxiety patients were observed using a randomized double-blind method. HAMA and self-made Traditional Chinese Medicine (TCM) symptom scales were utilized to assess symptoms before and after treatment, while the Treatment Emergent Symptom Scale (TESS) was applied to evaluate adverse reactions. Results of a 6-week observation period demonstrated that CJP could improve HAMA and self-made TCM symptom scale scores. Moreover, effects were significant at 2, 4, and 6 weeks, indicating a progressive effect compared with the placebo group. Importantly, the effective rate was 93.1% and no adverse reactions were observed [3].

CJP is composed of* Radix Bupleuri*,* Radix Scutellariae*,* Rhizoma Pinelliae Preparata*,* Radix Codonopsis*,* Radix Glycyrrhizae preparata*, keel,* Concha Ostreae*,* Concha Margaritifera Usta*,* Rhizoma Zingiberis Recens*, and* Fructus Jujubae*. Modern research has confirmed that saikoside, an important component of* Radix Bupleuri*, plays a role in cholinergic effects and regulates the digestive and nervous systems by suppressing cholinesterase [4]. Moreover,* Radix Bupleuri* decoction elicited improvements in physical symptoms, depression, and irritability [5, 6], which are mainly associated with regulation of hypothalamic-pituitary-adrenal axis hyperfunction and brain monoamine neurotransmitters [5]. Keel and* Concha Ostreae* have sedative and anticonvulsant effects [7], whereas total glycosides extracted from highly compatible* Radix Bupleuri*, keel, and* Concha Ostreae* can dramatically reduce hippocampal neuronal cell death rates [8], inhibit anxiety and depression, and improve rat behaviors [9].* Radix Codonopsis* can elicit resistance to fatigue and hypoxia and enhance immunity [10, 11]. Ginger propanol extract can have a cholagogic effect and inhibit 5-hydroxytryptamine. In addition to generating sedative, antihypertensive, and anticonvulsant effects,* Fructus Jujubae* can increase animal weight, enhance muscle strength, and evoke a certain antagonistic effect on 5-hydroxytryptamine and histamine. However, previous research has mainly focused on the role and effect of CJP on depression; indeed, to our knowledge, few studies have examined the effect of CJP on anxiety. In this study, we explored the mechanism underlying the antianxiety effects of CJP by observing effects in a rat model of anxiety disorder.

## 2. Materials and Methods

### 2.1. Materials

Clean male Sprague-Dawley rats were provided by the Animal Experimental Center of Nanjing University of Chinese Medicine of China [License number: SCXK (Su) 2013-0003]. All rats were acclimated for 1 week. Forty rats weighing 200 ± 20 g with similar behaviors, as screened by open-field test, were selected and randomly assigned to control (no treatment or anxiety model induction) and anxiety model groups: model (untreated), citalopram, low-dose CJP, and high-dose CJP. All procedures were approved by the Animal Ethics Committee of Lianyungang Affiliated Hospital, Nanjing University of Chinese Medicine (Nanjing, China).

CJP was provided by the Manufacturing Laboratory, Lianyungang Affiliated Hospital, Nanjing University of Chinese Medicine. Drug preparation: (1)* Radix Bupleuri*,* Radix Scutellariae*,* Radix Glycyrrhizae preparata*, keel,* Concha Ostreae*,* Concha Margaritifera Usta*,* Rhizoma Zingiberis Recens*, and* Fructus Jujubae* were decocted twice with water: first for 2 h and then for 1 h. The filtrate was mixed and concentrated to a moderate amount. (2)* Rhizoma Pinelliae Preparata* and* Radix Codonopsis* were triturated into powder, which was mixed with the above-described concentrated solution. A bolus was prepared, dried, and stored in bottles (No. Z04000043, 60 g/bottle). Citalopram (No. Z566665803, 20 mg/pill, 14 pills/box) was purchased from Xi'an Janssen Pharmaceutical (Xi'an, China).

### 2.2. Methods

#### 2.2.1. Model Establishment

Rat models of anxiety disorder were established using empty bottle stimulation in accordance with a previous study [12]. Briefly, rats in all groups except the control group were given access to regular drinking water only twice a day at 9:00—9:10 and 21:00—21:10 for 1 week. This entrainment was followed by a stress test: uncertain empty bottle stimulation was performed once for the above-described time period (i.e., rats could not drink water once) for 2 consecutive weeks [13–15] (Table 1).

#### 2.2.2. Drug Administration

One day after model establishment, rat appearance [16], weight [17], and open-field behaviors [18] were measured, and administration was carried out. Administration dosages for rats were 1620 mg/kg for the low-dose CJP group (equivalent to the dosage commonly used in adults), 3240 mg/kg for the high-dose CJP group (equivalent to a double dosage in adults), and 1.8 mg/kg citalopram hydrobromide. Medicines were intragastrically administered according to weight (1 mL/100 g) once daily. For untreated control and model groups, an equal volume of physiological saline was administered daily. Administration was conducted from 8:00 to 12:00. Thirty days later, rat behaviors were evaluated. Subsequently, rats were decapitated and the hippocampus was obtained.

### 2.3. Behavioral Indices

#### 2.3.1. General Observation of Rats

General appearance included hair, color, body posture, mental state, activity, color of the auricle, and resistance to restraint stress. Rats were weighed before and after administration.

#### 2.3.2. Open-Field Test

The open-field test is a widely used model to evaluate anxious behavior in animals [19, 20]. A self-made open box was used; the inner wall and undersurface of the cardboard box (80 cm × 80 cm × 80 cm) were covered with black oil paper and fixed with adhesive tape. The ground was equally divided into 25 squares with chalk. Rats were placed in the center of an open-field box to observe their horizontal and vertical movements. The frequency of crossing the undersurface squares was used to measure horizontal movement, and the frequency of being upright on hind limbs was used to measure vertical movement. The detection time was 5 min and each rat was examined once. Scoring criterion for horizontal movement: half of the body entering the other square was scored as 1. Scoring criterion of vertical movement: forelimbs were 1 cm above the ground; standing up on the hind limbs once was scored as 1.

#### 2.3.3. Specimen Extraction and Index Determination

Rats were intraperitoneally injected with 10% chloral hydrate (4 mL/100 g) and decapitated. Hippocampi (approximately 15 mg) were obtained, cut, and triturated. Approximately 500 *μ*L of physiological saline was added and mixed, followed by centrifugation at 5000 rpm for 10 min. After removing the supernatant, samples were stored at −80°C.

#### 2.3.4. Monoamine Neurotransmitter Detection

Blank and standard wells were set, and 10 *μ*L of samples (final dilution of the sample was 5-fold) was added to the bottom of the microplate. After lightly shaking, 50 *μ*L of standard preparation and 100 *μ*L of detected sample were added to the reaction wells. Biotin-labeled antibody (50 *μ*L) was immediately added, and a cover was placed on the plate. After lightly shaking and mixing, samples were incubated at 37°C for 45 min. The liquid in the well was discarded, and cleaning solution was added to each well for a 30-second shaking. The cleaning solution was discarded and the well was dried with absorbent paper. After four washes, 100 *μ*L of streptavidin-horseradish peroxidase was added to each well, and then the plate was lightly shaken and incubated at 37°C for 30 min, followed by four washes as above. Substrates A and B (50 *μ*L of each) were added to each well, then the plate was lightly shaken, mixed, and incubated at 37°C for 5 min in the dark. Microplates were then taken out and the reaction was rapidly terminated by adding 50 *μ*L of stop buffer.

#### 2.3.5. Detection of MR and GR mRNA Expression

Equipment and kits were as follows: cDNA First-Strand Synthesis Kit, Tap DNA Polymerase, Regular Agarose G10, polymerase chain reaction (PCR) cycler, and a nucleic acid electrophoresis apparatus. In accordance with the manufacturer's instructions, one-step RT-PCR was used to measure mRNA expression: MR mRNA upstream 3′—AAC AAA ATG CCC CAC GGT TA—5′ (20 bp), downstream: 3′—GGG ACG ATG CAA TGG ACT GT—5′ (20 bp); GR mRNA upstream 3′—GGA TTT CCA GAG CCC ACC AT—5′ (20 bp), downstream 3′—CAT TCC TGA TGG TCA CCT CG—5′ (20 bp).

### 2.4. Statistical Analysis

Data were analyzed using SPSS 19.0 software. Measurement data are expressed as mean ± SD. Multivariate analysis of variance was used to test the significance of group differences. Hypotheses were tested using a two-sided test. Values of* P* < 0.05 were considered statistically significant. Values of* P* < 0.01 were considered remarkably statistically significant. 

## 3. Results

### 3.1. General Conditions

After model establishment, rats in the control group showed good spirit, quick actions, bright and clean hair, and a normal diet. Rats in the model, citalopram, low-dose CJP, and high-dose CJP groups presented listlessness, messy hair, slow responses, violent resistance, screaming, and struggling. In the citalopram, low-dose CJP, and high-dose CJP groups, calls were soft, resistance and confrontation were weak, and the frequencies of breaking free and biting were reduced. In the model group, the reaction to restraint was intense, including struggling, biting the cage, braying, and trying to break free. In addition, rats in the model group had loose stools. Rats in control, citalopram, low-dose CJP, and high-dose CJP groups had moderate stool shapes.

### 3.2. Changes in Rat Weight

**Figure 1:**
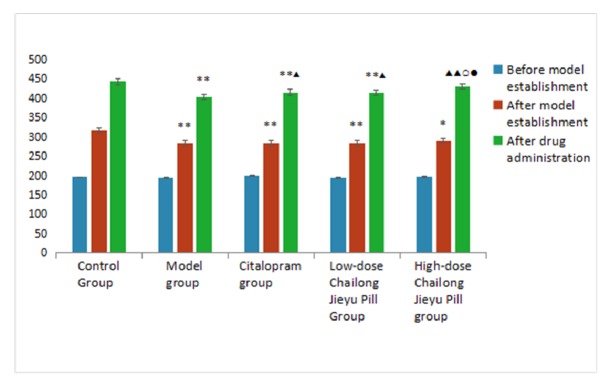
Rat body weight changes in each group. ^*∗*^P < 0.05, ^*∗∗*^P < 0.01, versus blank control group; ^▲^P < 0.05, ^▲▲^P < 0.01, versus model group; ^○^P < 0.05, citalopram group; ^●^P < 0.05, versus low-dose Chailong Jieyu Pill group.

The weight of rats was lower in model, citalopram, low-dose CJP, and high-dose CJP groups compared with the control group (*P* < 0.05), indicating successful model establishment. The weight of rats in citalopram, low-dose CJP, and high-dose CJP groups was increased compared with the model group (*P* < 0.01,* P* < 0.05). There was no difference in the weight change of rats in low-dose CJP and citalopram groups (*P* > 0.05). The weight of rats in the high-dose CJP group was increased compared with low-dose CJP and citalopram groups (*P* < 0.05) and similar to the control group (*P* > 0.05; Table 2, Figure 1).

### 3.3. Open-Field Test

**Figure 2:**
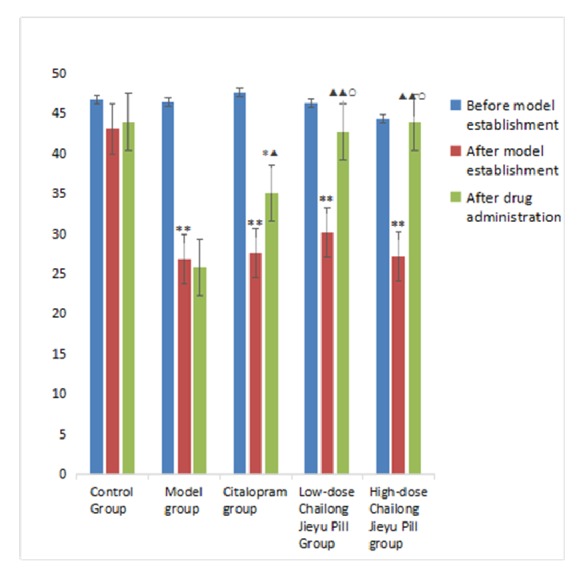
Comparison of horizontal movements at different time points in rats from each group. ^*∗*^P < 0.05, ^*∗∗*^P < 0.01, versus blank control group; ^▲^P < 0.05, ^▲▲^P < 0.01, versus model group; ^○^P < 0.05, versus citalopram group.

**Figure 3:**
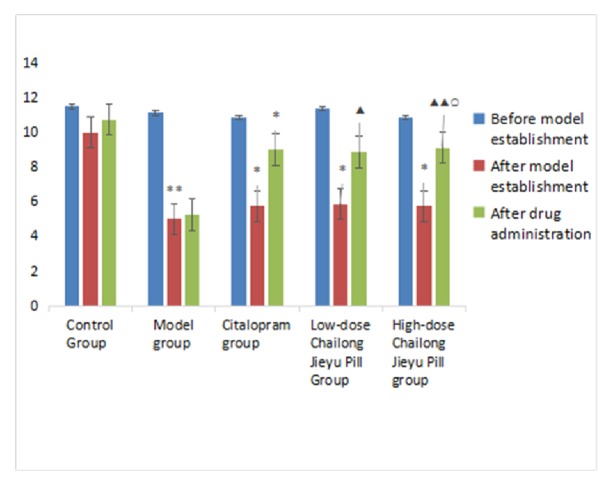
Comparison of vertical movements at different time points in rats from each group. ^*∗*^P < 0.05, ^*∗∗*^P < 0.01, versus blank control group; ^▲^P < 0.05, ^▲▲^P < 0.01, versus model group; ^○^P < 0.05, versus citalopram group.

Scores for horizontal and vertical movements were significantly lower in model, citalopram, low-dose CJP, and high-dose CJP groups compared with the control group (*P* < 0.05). After administration, horizontal movement scores were higher in the citalopram group than in control and model groups (*P *< 0.05), while vertical movement scores were more improved than in the control group (*P* < 0.05). Horizontal movement scores were significantly higher in the low-dose CJP group than in model and citalopram groups* (P* < 0.01,* P* < 0.05), and vertical movement scores were also increased compared with the model group (*P* < 0.05). Scores for horizontal and vertical movements in the high-dose CJP group were significantly higher than in the model group* (P* < 0.01,* P* < 0.05) and were improved compared with the citalopram group (P < 0.05). Scores of horizontal and vertical movements were not significantly different between low-dose and high-dose CJP groups (*P* > 0.05; Table 3, Figures 2 and 3).

### 3.4. Monoamine Neurotransmitters

**Figure 4:**
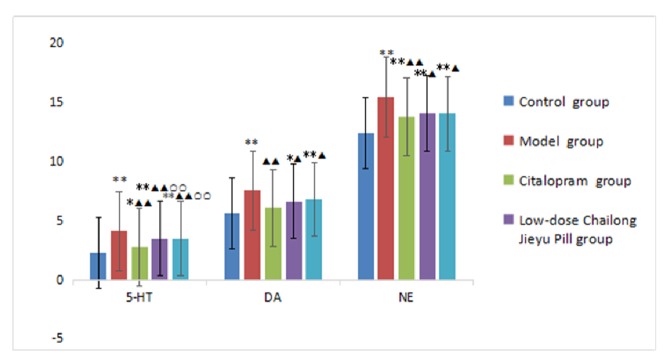
Comparison of monoamine neurotransmitters in the brains of rats in each group. ^*∗*^P < 0.05, ^*∗∗*^P < 0.01, versus blank control group; ^▲^P < 0.05, ^▲▲^P < 0.01, versus model group; ^○○^P < 0.01, citalopram group.

5-Hydroxytryptamine, dopamine, and norepinephrine contents were reduced in each administration group, with levels in the citalopram group being lower than in the model group (*P* < 0.01), but still higher than in the control group. Contents of all three neurotransmitters were higher in the low-dose CJP group compared with the citalopram group. Interestingly, 5-hydroxytryptamine contents were significantly different (*P* < 0.01), but dopamine and norepinephrine contents were not (*P* > 0.05) between low-dose CJP and citalopram groups or between high-dose CJP and citalopram groups. 5-Hydroxytryptamine, dopamine, and norepinephrine contents were not significantly different between low-dose and high-dose CJP groups (*P* > 0.05; Table 4, Figure 4).

### 3.5. MR and GR mRNA Expression

**Figure 5:**
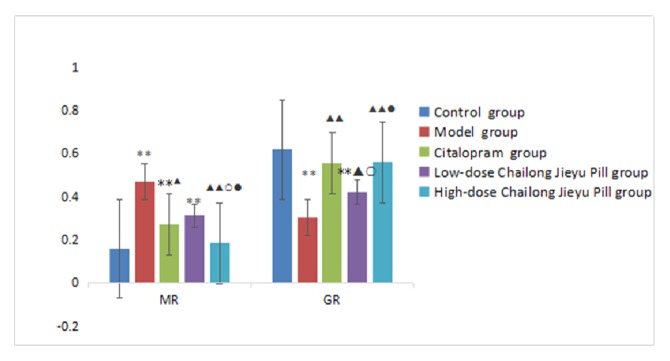
Comparison of MR and GR gene expression in brains of rats in each group. ^*∗*^P < 0.01, versus blank control group; ^▲^P < 0.05, ^▲▲^P < 0.01, versus model group; ^○^P < 0.05, versus citalopram group; ^●^P < 0.05, versus low-dose Chailong Jieyu Pill group. MR, mineralocorticoid receptor; GR, glucocorticoid receptor.

MR mRNA expression was upregulated, but GR mRNA expression was significantly downregulated in the hippocampi of rats in the model group compared with control group rats (*P* < 0.01). After administration, MR mRNA expression was downregulated (*P* < 0.05), but GR mRNA expression was upregulated (*P* < 0.01) in the citalopram group compared with the model group. MR mRNA expression was not significantly downregulated in the low-dose CJP group. Moreover, MR mRNA expression was not significantly different between low-dose CJP and citalopram groups (P > 0.05). GR mRNA expression in the low-dose CJP group was significantly upregulated (*P* < 0.05); however, this level remained significantly lower than observed in the citalopram group (*P* < 0.05). In the high-dose CJP group, MR mRNA expression was significantly downregulated, while GR mRNA expression was upregulated (*P* < 0.01). Downregulation of MR mRNA expression was enhanced in the high-dose CJP group compared with citalopram (*P* < 0.05) and low-dose CJP (*P* < 0.05; Table 5, Figure 5) groups.

## 4. Discussion

In the present study, we used empty bottle stimulation to establish an anxiety model in rats. Empty bottle stimulation is a mature, easily operated, and generally accepted method for the preparation of anxious animals. The method of empty bottle stimulation used to establish our model of anxiety disorder was first proposed by Izquierdo et al. [21]. Briefly, rats are trained to drink water at regular times to produce a conditioned reflex, and then empty water bottles are irregularly placed in cages to simulate anxiety responses within rats. General conditions, weight changes, and open-field test results demonstrated that rat weight slowly increased (*P* < 0.01) and scores for horizontal and vertical movement were reduced (*P* < 0.05) in the model group, indicating that rats presented anxious behaviors and the model was successfully established. The weight of rats in the low-dose CJP group was increased and identical to animals in the citalopram group (*P* > 0.05), but still lower than observed in the control group (*P* < 0.01). Rat weight was increased in the high-dose CJP group compared with the citalopram group (*P *< 0.01) and identical to that of the control group (*P* > 0.05). This may be associated with the ability of CJP to disperse depressed liver energy, calm nerves with heavy material, invigorate the spleen and stomach, relieve tension, improve anxiety state, and increase appetite. In the open-field test, horizontal movement scores were significantly increased (*P* < 0.01), and vertical movement scores were increased (*P* < 0.05) in the low-dose CJP group. Scores for horizontal and vertical movements were identical between low-dose CJP and control groups (*P* > 0.05). Scores for horizontal and vertical movements in the high-dose CJP group were increased (*P *< 0.01), better than observed in the citalopram group (*P* < 0.05), and identical to the control group (*P* > 0.05). Vertical movement indicates active behavior, which decreases with anxiety disorder. Vertical movement scores of rats were lower than horizontal movement scores after modeling. Therefore, elevated vertical movement scores are more meaningful than elevated horizontal movement scores. Improvement in the vertical movement was not significant in the citalopram group (*P* > 0.05), but was significant in both low-dose and high-dose CJP groups (*P* < 0.05). Thus, it is evident that CJP has significant antianxiety effects in anxious rats.

The current diagnosis of anxiety is still based on clinical manifestations and scales as the main diagnostic criteria, as a result of inaccurate etiology and lack of relevant objective laboratory indicators. While the diagnosis of anxiety is mainly based on symptoms [22], the cause of anxiety remains unclear. However, imbalances in various neurotransmitters including gamma-aminobutyric acid (GABA) and the monoamines dopamine, norepinephrine, and serotonin, have been implicated as important mechanisms leading to anxiety [23]. The incidence of anxiety disorders is associated with excessive release of monoamine neurotransmitters in the brain. Indeed, drugs that reduce 5-hydroxytryptamine, dopamine, and norepinephrine exert an anxiolytic effect [24]. An increased 5-hydroxytryptamine concentration is directly proportional to anxiety attacks [25]. Neuroleptic agents can alleviate clinical manifestations of anxiety disorder and reduce dopaminergic transmission, suggesting a potentially important role for the dopaminergic system in pathogenesis of anxiety disorders [26]. While the role of norepinephrine in anxiety disorders is not well understood, research examining cerebrospinal fluid, blood, and urine observed noradrenergic activity during an anxiety attack [27]. Low-dose and high-dose CJP remarkably reduced monoamine neurotransmitter contents in the rat hippocampus. However, the ability of CJP to reduce 5-hydroxytryptamine content was less than that of citalopram (*P* < 0.01), while the ability of these two treatments to decrease dopamine and norepinephrine contents were similar (*P* > 0.05). It is hypothesized that CJP exerted an antianxiety effect by reducing the release of monoamine neurotransmitters in the hippocampus. However, this is not exactly the case because the degree of reduced 5-hydroxytryptamine, which is closely related to anxiety, was significantly weaker than observed for citalopram, although the improvement of behavioral index was better than citalopram. These results suggest that the beneficial effects of CJP on anxiety are not limited to monoamine transmitters, the effects of CJP on the metabolites of these neurotransmitters need to be further investigated. Moreover, the effects of high-dose and low-dose CJP on monoamine neurotransmitters were not significantly different, suggesting that the effects of CJP on anxiety disorder were not significantly dose-dependent. While the reason for an absence of dose dependence is not yet clear, it may be due to the fact that the difference between high and low dosages was too small. Indeed, as a 1-fold higher dose of CJP was used in the present study, the effect of a 3- to 5-fold higher dose of CJP on anxiety needs to be further investigated.

Hippocampal neurons contain abundant MR and GR, with the balance between these two receptors playing an important role in neuronal excitability and stress responses [28]. MR content determines the level of cortisol, which is associated with fear, whereas GR inhibits hypothalamic-pituitary-adrenal axis overreaction under stress [29]. Patients with chronic emotional stimuli exhibit sustained low cortisol responses, hippocampal imaging changes, and enhanced negative feedback control of the hypothalamic-pituitary-adrenal axis [30]. Few studies have examined the effect of Chinese medicine preparation on MR and GR expression. In this study, we observed the effects of CJP on MR and GR mRNA expression, we found that MR downregulation was not significant in the low-dose CJP group (*P* > 0.05), but MR mRNA expression was not significantly different between the low-dose CJP and citalopram groups (*P* > 0.05). GR mRNA expression in the low-dose CJP group was significantly upregulated (*P* < 0.05), but remained significantly less than observed in the citalopram group (*P* < 0.05). In the high-dose CJP group, MR mRNA expression was significantly downregulated, but GR mRNA expression was upregulated (*P* < 0.01). The downregulation of MR mRNA expression was enhanced in the high-dose CJP group compared with citalopram (*P* < 0.05) and low-dose CJP (*P* < 0.05) groups, but similar to the control group (*P* > 0.05). Taken together, CJP could obviously regulate MR and GR mRNA expression in a dose-dependent manner. This effect was enhanced in the high-dose CJP group compared with the citalopram group and was close to normal. This may be the reason the regulatory effect of CJP on 5-hydroxytryptamine is less than citalopram, but the improvement on rat behaviors is better. Our results suggest that CJP has a moderate effect on regulating neurotransmitters and a significant effect on regulating gene expression, which are different from that of citalopram. Moreover, MR and GR mRNA expression are sensitive to CJP treatment, although the exact mechanism remains to be elucidated.

## 5. Conclusions

Our results verified that CJP could remarkably improve anxious behavior, increase weight, reduce hippocampal monoamine neurotransmitters, and regulate MR and GR mRNA expression. Nevertheless, we still poorly understood why the regulatory effect of low-dose CJP on MR and GR mRNA expression was not significant, but the effect of high-dose CJP was very significant. This requires further investigation.

## Figures and Tables

**Table 1:** Empty bottle stimulus schedule.

Time	1	2	3	4	5	6	7	8	9	10	11	12	13	14	15	16	17	18	19	20	21
9:00	ES	ES	ES	ES	ES	ES	N	N	N	ES	N	N	ES	N	ES	ES	N	ES	N	N	ES
21:00	ES	ES	ES	ES	ES	ES	ES	ES	ES	N	ES	ES	N	ES	N	N	ES	N	ES	ES	N

Note: ES: water supply; N: empty bottle.

**Table 2:** Comparison of weight changes of rats in each group (mean ± SD, *n* = 40, g).

Time (day)	Blank control group	Model group	Citalopram group	Low-dose Chailong Jieyu Pill group	High-dose Chailong Jieyu Pill group
0	196.38±5.24	195.38±6.52	199.63±5.37	195.13±10.45	197.38±5.26
22	317.38±28.02	283.88±12.35^*∗∗*^	284.63±11.66^*∗∗*^	284.50±9.38^*∗∗*^	289.38±22.96^*∗*^
52	443.25±20.39	402.75±12.03^*∗∗*^	415.75±9.71^*∗∗*▲^	414.38±9.15^*∗∗*▲^	431.13±13.40^▲▲○●^

^*∗*^P < 0.05, ^*∗∗*^P < 0.01, versus blank control group; ^▲^P < 0.05, ^▲▲^P < 0.01, versus model group;^ ○^P < 0.05, citalopram group; ^●^P < 0.05, versus low-dose Chailong Jieyu Pill group.

**Table 3:** Comparison of open-field test results at different time points in each group (mean ± SD, score,* n* = 40).

	Group	Before modeling	After modeling	After administration
Horizontal movement	Blank control	46.75±6.80	43.13±5.91	44.00±5.95
	Model	46.50±6.55	26.88±5.91^*∗∗*^	25.88±7.70
	Citalopram	47.63±11.51	27.63±7.23^*∗∗*^	35.13±8.60^*∗*▲^
	Low-dose Chailong Jieyu Pill	46.38±12.20	30.13±8.01^*∗∗*^	42.75±3.96^▲▲○^
	High-dose Chailong Jieyu Pill	44.38±9.12	27.13±7.26^*∗∗*^	43.88±7.26^▲▲○^
Vertical movement	Blank control	11.50±3.21	10.00±3.46	10.75±4.27
	Model	11.13±2.70	5.00±2.27^*∗∗*^	5.25±2.38
	Citalopram	10.88±4.36	5.75±3.11^*∗*^	6.88±2.30^*∗*^
	Low-dose Chailong Jieyu Pill	11.38±4.10	5.88±2.90^*∗*^	9.25±3.88^▲^
	High-dose Chailong Jieyu Pill	0.88±3.09	5.75±3.92^*∗*^	9.75±2.76^▲▲○^

^*∗*^P < 0.05, ^*∗∗*^P < 0.01, versus blank control group; ^▲^P < 0.05, ^▲▲^P < 0.01, versus model group; ^○^P < 0.05, versus citalopram group.

**Table 4:** Comparison of monoamine neurotransmitter contents in rats of each group (mean ± SD, ng/mL, *n *= 40).

Group	5-Hydroxytryptamine	Dopamine	Norepinephrine
Blank control	2.285±0.445	5.578±0.618	12.44±0.638
Model	4.115±0.459^*∗∗*^	7.568±0.438^*∗∗*^	15.462±1.157^*∗∗*^
Citalopram	2.754±0.225^*∗*▲▲^	6.084±0.528^▲▲^	13.790±0.986^*∗∗*▲▲^
Low-dose Chailong Jieyu Pill	3.456±0.284^*∗∗*▲▲○○^	6.615±1.171^*∗*▲^	14.077±0.829^*∗∗*▲^
High-dose Chailong Jieyu Pill	3.494±0.106^*∗∗*▲▲○○^	6.781±0.863^*∗∗*▲^	14.045±1.263^*∗∗*▲^

^*∗*^P < 0.05, ^*∗∗*^P < 0.01, versus blank control group; ^▲^P < 0.05, ^▲▲^P < 0.01, versus model group; ^○○^P < 0.01, citalopram group.

**Table 5:** Comparison of MR and GR mRNA expression in the brain of rats from each group (mean ± SD, n = 40).

Group	MR	GR
Blank control	0.160±0.052	0.618±0.121
Model	0.470±0.186^*∗*^	0.303±0.080^*∗*^
Citalopram	0.272±0.089^*∗*▲^	0.557±0.103^▲▲^
Low-dose Chailong Jieyu Pill	0.312±0.107^*∗*^	0.422±0.103^*∗*▲○^
High-dose Chailong Jieyu Pill	0.185±0.058^▲▲○●^	0.562±0.107^▲▲●^

^*∗*^P < 0.01, versus blank control group; ^▲^P < 0.05, ^▲▲^P < 0.01, versus model group; ^○^P < 0.05, versus citalopram group; ^●^P < 0.05, versus low-dose Chailong Jieyu Pill group. MR, mineralocorticoid receptor; GR, glucocorticoid receptor.

## Data Availability

The data used to support the findings of this study are available from the corresponding author upon request.
